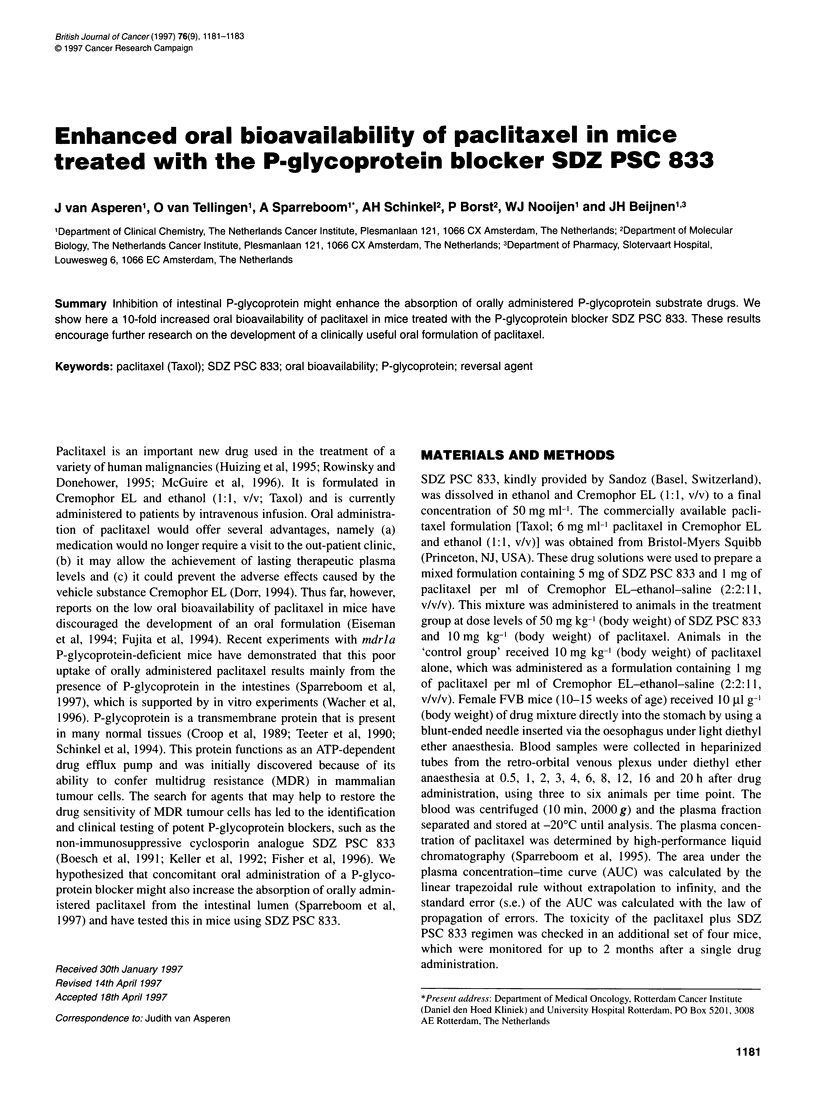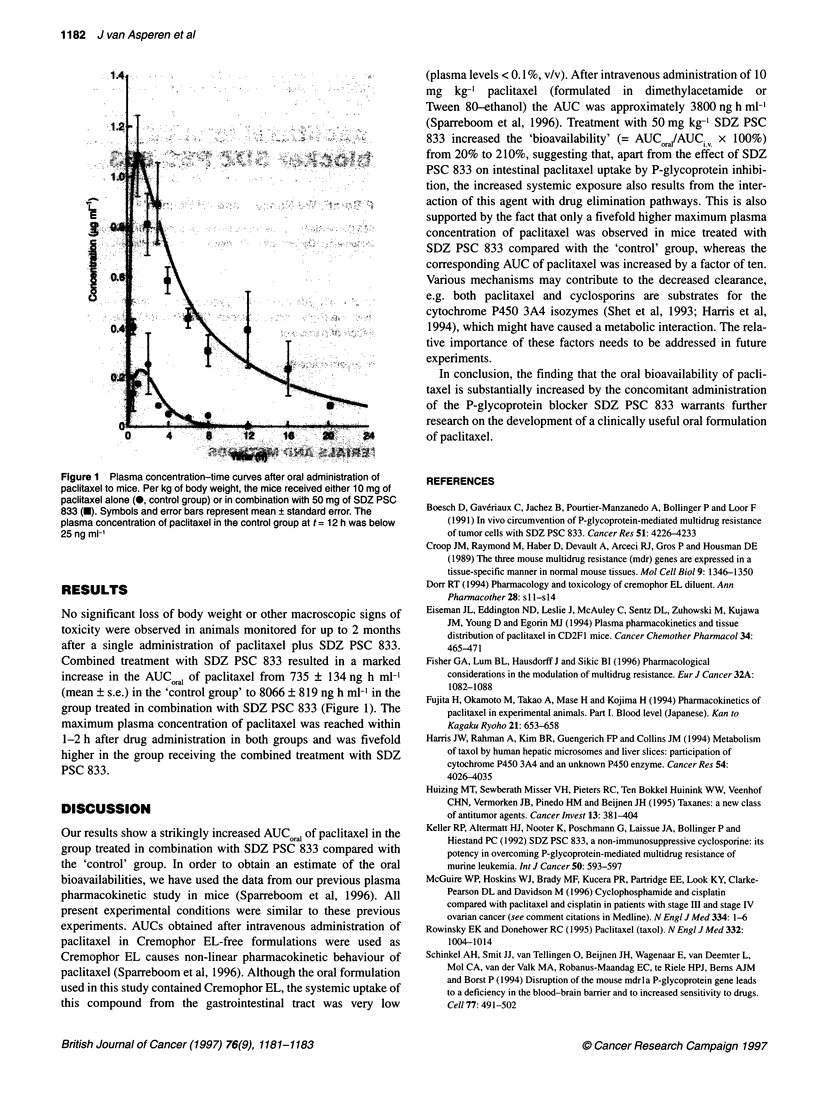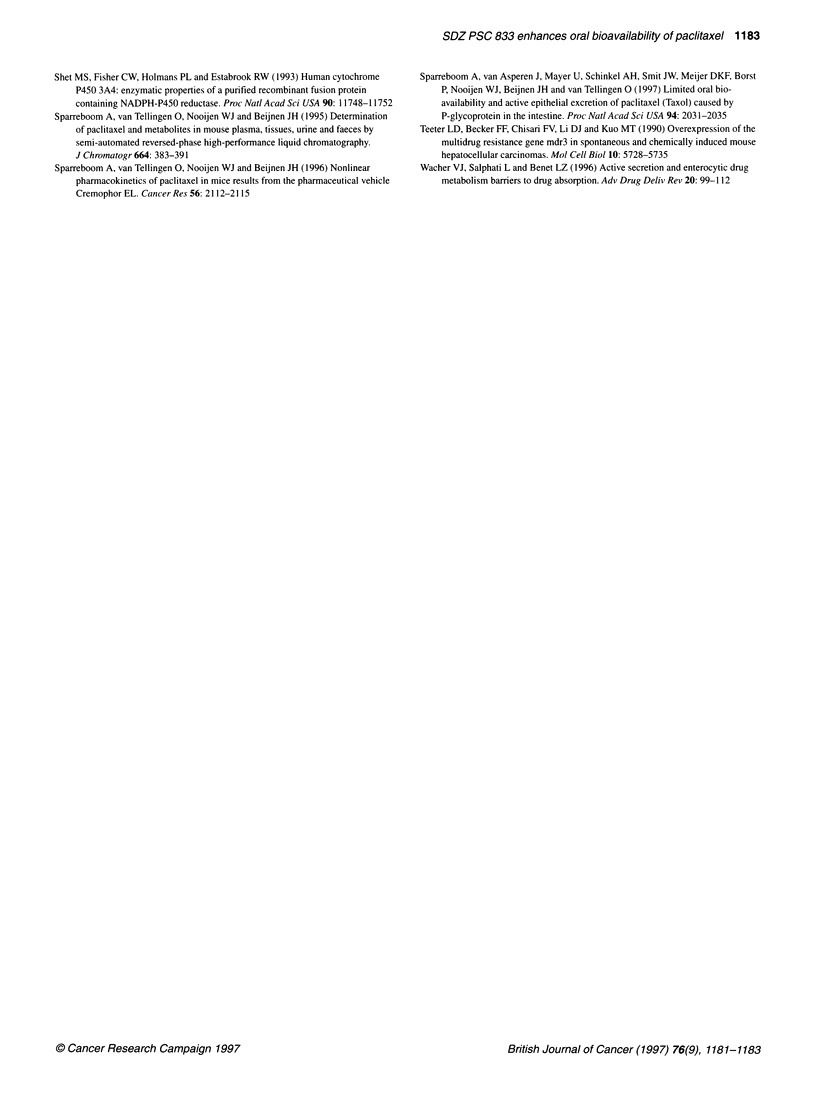# Enhanced oral bioavailability of paclitaxel in mice treated with the P-glycoprotein blocker SDZ PSC 833.

**DOI:** 10.1038/bjc.1997.530

**Published:** 1997

**Authors:** J. van Asperen, O. van Tellingen, A. Sparreboom, A. H. Schinkel, P. Borst, W. J. Nooijen, J. H. Beijnen

**Affiliations:** Department of Clinical Chemistry, The Netherlands Cancer Institute, Amsterdam.

## Abstract

Inhibition of intestinal P-glycoprotein might enhance the absorption of orally administered P-glycoprotein substrate drugs. We show here a 10-fold increased oral bioavailability of paclitaxel in mice treated with the P-glycoprotein blocker SDZ PSC 833. These results encourage further research on the development of a clinically useful oral formulation of paclitaxel.


British Journal of Cancer (1997) 76(9), 1181–1183
© 1997 Cancer Research Campaign

### Enhanced oral bioavailability of paclitaxel in mice treated with the P-glycoprotein blocker SDZ PSC 833

J van Asperen[1], O van Tellingen[1], A Sparreboom[1*], AH Schinkel[2], P Borst[2], WJ Nooijen[1] and JH Beijnen[1,3]

[1]Department of Clinical Chemistry, The Netherlands Cancer Institute, Plesmanlaan 121, 1066 CX Amsterdam, The Netherlands; [2]Department of Molecular Biology, The Netherlands Cancer Institute, Plesmanlaan 121, 1066 CX Amsterdam, The Netherlands; [3]Department of Pharmacy, Slotervaart Hospital, Louwesweg 6, 1066 EC Amsterdam, The Netherlands

**Summary** Inhibition of intestinal P-glycoprotein might enhance the absorption of orally administered P-glycoprotein substrate drugs. We show here a 10-fold increased oral bioavailability of paclitaxel in mice treated with the P-glycoprotein blocker SDZ PSC 833. These results encourage further research on the development of a clinically useful oral formulation of paclitaxel.

**Keywords:** paclitaxel (Taxol); SDZ PSC 833; oral bioavailability; P-glycoprotein; reversal agent

Paclitaxel is an important new drug used in the treatment of a variety of human malignancies (Huizing et al, 1995; Rowinsky and Donehower, 1995; McGuire et al, 1996). It is formulated in Cremophor EL and ethanol (1:1, v/v; Taxol) and is currently administered to patients by intravenous infusion. Oral administration of paclitaxel would offer several advantages, namely (a) medication would no longer require a visit to the out-patient clinic, (b) it may allow the achievement of lasting therapeutic plasma levels and (c) it could prevent the adverse effects caused by the vehicle substance Cremophor EL (Dorr, 1994). Thus far, however, reports on the low oral bioavailability of paclitaxel in mice have discouraged the development of an oral formulation (Eiseman et al, 1994; Fujita et al, 1994). Recent experiments with *mdr1a* P-glycoprotein-deficient mice have demonstrated that this poor uptake of orally administered paclitaxel results mainly from the presence of P-glycoprotein in the intestines (Sparreboom et al, 1997), which is supported by in vitro experiments (Wacher et al, 1996). P-glycoprotein is a transmembrane protein that is present in many normal tissues (Croop et al, 1989; Teeter et al, 1990; Schinkel et al, 1994). This protein functions as an ATP-dependent drug efflux pump and was initially discovered because of its ability to confer multidrug resistance (MDR) in mammalian tumour cells. The search for agents that may help to restore the drug sensitivity of MDR tumour cells has led to the identification and clinical testing of potent P-glycoprotein blockers, such as the non-immunosuppressive cyclosporin analogue SDZ PSC 833 (Boesch et al, 1991; Keller et al, 1992; Fisher et al, 1996). We hypothesized that concomitant oral administration of a P-glycoprotein blocker might also increase the absorption of orally administered paclitaxel from the intestinal lumen (Sparreboom et al, 1997) and have tested this in mice using SDZ PSC 833.

*Received 30th January 1997*
*Revised 14th April 1997*
*Accepted 18th April 1997*

*Correspondence to:* Judith van Asperen

### MATERIALS AND METHODS

SDZ PSC 833, kindly provided by Sandoz (Basel, Switzerland), was dissolved in ethanol and Cremophor EL (1:1, v/v) to a final concentration of 50 mg $ml^{-1}$. The commercially available paclitaxel formulation [Taxol; 6 mg $ml^{-1}$ paclitaxel in Cremophor EL and ethanol (1:1, v/v)] was obtained from Bristol-Myers Squibb (Princeton, NJ, USA). These drug solutions were used to prepare a mixed formulation containing 5 mg of SDZ PSC 833 and 1 mg of paclitaxel per ml of Cremophor EL–ethanol–saline (2:2:11, v/v/v). This mixture was administered to animals in the treatment group at dose levels of 50 mg $kg^{-1}$ (body weight) of SDZ PSC 833 and 10 mg $kg^{-1}$ (body weight) of paclitaxel. Animals in the 'control group' received 10 mg $kg^{-1}$ (body weight) of paclitaxel alone, which was administered as a formulation containing 1 mg of paclitaxel per ml of Cremophor EL–ethanol–saline (2:2:11, v/v/v). Female FVB mice (10–15 weeks of age) received 10 µl $g^{-1}$ (body weight) of drug mixture directly into the stomach by using a blunt-ended needle inserted via the oesophagus under light diethyl ether anaesthesia. Blood samples were collected in heparinized tubes from the retro-orbital venous plexus under diethyl ether anaesthesia at 0.5, 1, 2, 3, 4, 6, 8, 12, 16 and 20 h after drug administration, using three to six animals per time point. The blood was centrifuged (10 min, 2000 *g*) and the plasma fraction separated and stored at –20°C until analysis. The plasma concentration of paclitaxel was determined by high-performance liquid chromatography (Sparreboom et al, 1995). The area under the plasma concentration–time curve (AUC) was calculated by the linear trapezoidal rule without extrapolation to infinity, and the standard error (s.e.) of the AUC was calculated with the law of propagation of errors. The toxicity of the paclitaxel plus SDZ PSC 833 regimen was checked in an additional set of four mice, which were monitored for up to 2 months after a single drug administration.

**Present address*: Department of Medical Oncology, Rotterdam Cancer Institute (Daniel den Hoed Kliniek) and University Hospital Rotterdam, PO Box 5201, 3008 AE Rotterdam, The Netherlands

**Figure 1** Plasma concentration–time curves after oral administration of paclitaxel to mice. Per kg of body weight, the mice received either 10 mg of paclitaxel alone (●, control group) or in combination with 50 mg of SDZ PSC 833 (■). Symbols and error bars represent mean ± standard error. The plasma concentration of paclitaxel in the control group at $t = 12$ h was below 25 ng ml$^{-1}$

### RESULTS

No significant loss of body weight or other macroscopic signs of toxicity were observed in animals monitored for up to 2 months after a single administration of paclitaxel plus SDZ PSC 833. Combined treatment with SDZ PSC 833 resulted in a marked increase in the $AUC_{oral}$ of paclitaxel from 735 ± 134 ng h ml$^{-1}$ (mean ± s.e.) in the 'control group' to 8066 ± 819 ng h ml$^{-1}$ in the group treated in combination with SDZ PSC 833 (Figure 1). The maximum plasma concentration of paclitaxel was reached within 1–2 h after drug administration in both groups and was fivefold higher in the group receiving the combined treatment with SDZ PSC 833.

### DISCUSSION

Our results show a strikingly increased $AUC_{oral}$ of paclitaxel in the group treated in combination with SDZ PSC 833 compared with the 'control' group. In order to obtain an estimate of the oral bioavailabilities, we have used the data from our previous plasma pharmacokinetic study in mice (Sparreboom et al, 1996). All present experimental conditions were similar to these previous experiments. AUCs obtained after intravenous administration of paclitaxel in Cremophor EL-free formulations were used as Cremophor EL causes non-linear pharmacokinetic behaviour of paclitaxel (Sparreboom et al, 1996). Although the oral formulation used in this study contained Cremophor EL, the systemic uptake of this compound from the gastrointestinal tract was very low (plasma levels < 0.1%, v/v). After intravenous administration of 10 mg kg$^{-1}$ paclitaxel (formulated in dimethylacetamide or Tween 80–ethanol) the AUC was approximately 3800 ng h ml$^{-1}$ (Sparreboom et al, 1996). Treatment with 50 mg kg$^{-1}$ SDZ PSC 833 increased the 'bioavailability' (= $AUC_{oral}/AUC_{i.v.} \times 100\%$) from 20% to 210%, suggesting that, apart from the effect of SDZ PSC 833 on intestinal paclitaxel uptake by P-glycoprotein inhibition, the increased systemic exposure also results from the interaction of this agent with drug elimination pathways. This is also supported by the fact that only a fivefold higher maximum plasma concentration of paclitaxel was observed in mice treated with SDZ PSC 833 compared with the 'control' group, whereas the corresponding AUC of paclitaxel was increased by a factor of ten. Various mechanisms may contribute to the decreased clearance, e.g. both paclitaxel and cyclosporins are substrates for the cytochrome P450 3A4 isozymes (Shet et al, 1993; Harris et al, 1994), which might have caused a metabolic interaction. The relative importance of these factors needs to be addressed in future experiments.

In conclusion, the finding that the oral bioavailability of paclitaxel is substantially increased by the concomitant administration of the P-glycoprotein blocker SDZ PSC 833 warrants further research on the development of a clinically useful oral formulation of paclitaxel.

### REFERENCES

Boesch D, Gavériaux C, Jachez B, Pourtier-Manzanedo A, Bollinger P and Loor F (1991) In vivo circumvention of P-glycoprotein-mediated multidrug resistance of tumor cells with SDZ PSC 833. *Cancer Res* **51**: 4226–4233

Croop JM, Raymond M, Haber D, Devault A, Arceci RJ, Gros P and Housman DE (1989) The three mouse multidrug resistance (mdr) genes are expressed in a tissue-specific manner in normal mouse tissues. *Mol Cell Biol* **9**: 1346–1350

Dorr RT (1994) Pharmacology and toxicology of cremophor EL diluent. *Ann Pharmacother* **28**: s11–s14

Eiseman JL, Eddington ND, Leslie J, McAuley C, Sentz DL, Zuhowski M, Kujawa JM, Young D and Egorin MJ (1994) Plasma pharmacokinetics and tissue distribution of paclitaxel in CD2F1 mice. *Cancer Chemother Pharmacol* **34**: 465–471

Fisher GA, Lum BL, Hausdorff J and Sikic BI (1996) Pharmacological considerations in the modulation of multidrug resistance. *Eur J Cancer* **32A**: 1082–1088

Fujita H, Okamoto M, Takao A, Mase H and Kojima H (1994) Pharmacokinetics of paclitaxel in experimental animals. Part I. Blood level (Japanese). *Kan to Kagaku Ryoho* **21**: 653–658

Harris JW, Rahman A, Kim BR, Guengerich FP and Collins JM (1994) Metabolism of taxol by human hepatic microsomes and liver slices: participation of cytochrome P450 3A4 and an unknown P450 enzyme. *Cancer Res* **54**: 4026–4035

Huizing MT, Sewberath Misser VH, Pieters RC, Ten Bokkel Huinink WW, Veenhof CHN, Vermorken JB, Pinedo HM and Beijnen JH (1995) Taxanes: a new class of antitumor agents. *Cancer Invest* **13**: 381–404

Keller RP, Altermatt HJ, Nooter K, Poschmann G, Laissue JA, Bollinger P and Hiestand PC (1992) SDZ PSC 833, a non-immunosuppressive cyclosporine: its potency in overcoming P-glycoprotein-mediated multidrug resistance of murine leukemia. *Int J Cancer* **50**: 593–597

McGuire WP, Hoskins WJ, Brady MF, Kucera PR, Partridge EE, Look KY, Clarke-Pearson DL and Davidson M (1996) Cyclophosphamide and cisplatin compared with paclitaxel and cisplatin in patients with stage III and stage IV ovarian cancer (*see* comment citations in Medline). *N Engl J Med* **334**: 1–6

Rowinsky EK and Donehower RC (1995) Paclitaxel (taxol). *N Engl J Med* **332**: 1004–1014

Schinkel AH, Smit JJ, van Tellingen O, Beijnen JH, Wagenaar E, van Deemter L, Mol CA, van der Valk MA, Robanus-Maandag EC, te Riele HPJ, Berns AJM and Borst P (1994) Disruption of the mouse mdr1a P-glycoprotein gene leads to a deficiency in the blood–brain barrier and to increased sensitivity to drugs. *Cell* **77**: 491–502

*© Cancer Research Campaign 1997*

Shet MS, Fisher CW, Holmans PL and Estabrook RW (1993) Human cytochrome P450 3A4: enzymatic properties of a purified recombinant fusion protein containing NADPH-P450 reductase. *Proc Natl Acad Sci USA* **90**: 11748–11752

Sparreboom A, van Tellingen O, Nooijen WJ and Beijnen JH (1995) Determination of paclitaxel and metabolites in mouse plasma, tissues, urine and faeces by semi-automated reversed-phase high-performance liquid chromatography. *J Chromatogr* **664**: 383–391

Sparreboom A, van Tellingen O, Nooijen WJ and Beijnen JH (1996) Nonlinear pharmacokinetics of paclitaxel in mice results from the pharmaceutical vehicle Cremophor EL. *Cancer Res* **56**: 2112–2115

Sparreboom A, van Asperen J, Mayer U, Schinkel AH, Smit JW, Meijer DKF, Borst P, Nooijen WJ, Beijnen JH and van Tellingen O (1997) Limited oral bioavailability and active epithelial excretion of paclitaxel (Taxol) caused by P-glycoprotein in the intestine. *Proc Natl Acad Sci USA* **94**: 2031–2035

Teeter LD, Becker FF, Chisari FV, Li DJ and Kuo MT (1990) Overexpression of the multidrug resistance gene mdr3 in spontaneous and chemically induced mouse hepatocellular carcinomas. *Mol Cell Biol* **10**: 5728–5735

Wacher VJ, Salphati L and Benet LZ (1996) Active secretion and enterocytic drug metabolism barriers to drug absorption. *Adv Drug Deliv Rev* **20**: 99–112

© Cancer Research Campaign 1997